# Endourologic and Open Ureterolithotomy and Common Sheath Reimplant for Large Bladder and Distal Ureteral Calculi

**DOI:** 10.1089/cren.2016.0098

**Published:** 2016-11-01

**Authors:** Madeline Cancian, Joseph Brito, Joseph Renzulli, Gyan Pareek

**Affiliations:** Department of Urology, Minimally Invasive Urology Institute, Brown University, Providence, Rhode Island.

**Keywords:** ureteroscopy, urolithiasis, duplicate ureter, lithotripsy

## Abstract

A twenty-eight-year-old female with a history of suprapubic pain and recurrent urinary tract infections presents for urology referral with a kidney, ureter, and bladder radiograph showing a 4.4 cm bladder calculus and 6.5 cm distal left ureteral stone. She underwent effective cystolitholapaxy of the bladder stone. Endourologic attempt (left ureteroscopy) was unsuccessful because of ureteral stone burden. Findings at ureteroscopy revealed a duplicated system on the left with the lower pole moiety joining just proximal to the ureteral orifice. The stone was found to be in the upper pole moiety ureter. An open ureterolithotomy was performed with intraoperative ureteroscopic laser lithotripsy and common sheath ureteral reimplant. Furthermore, a previously placed stent was found to be encrusted at the time of the ureterolithotomy. Effective ureteroscopy and lasering were performed through the ureterotomy up to the renal pelvis of the upper pole ureter.

## Clinical History

**FIG. 1.**
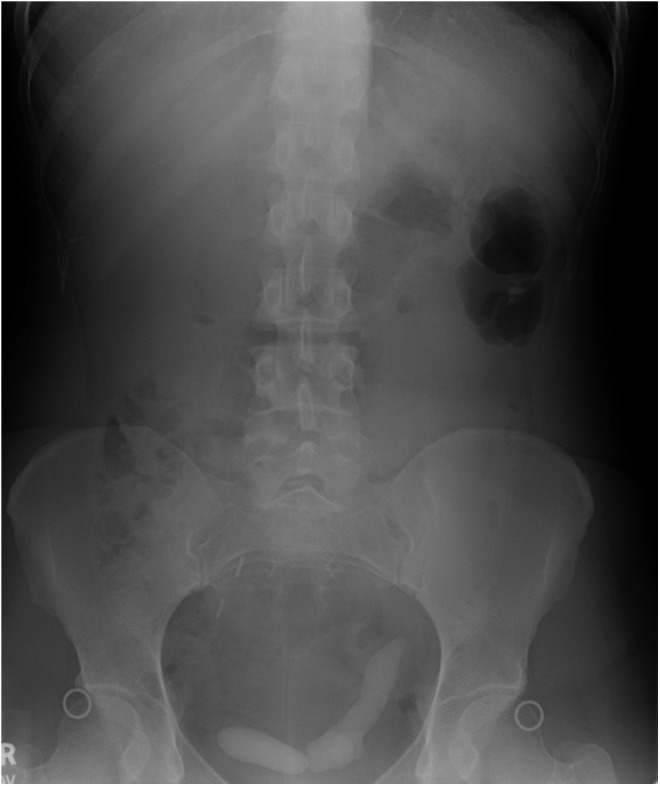
Kidney, ureter, and bladder radiograph from initial presentation.

A 28-year-old white female with a history of substance abuse on methadone and with recurrent urinary tract infections (UTIs) and nephrolithiasis not requiring intervention presented to the emergency department (ED) with suprapubic pain. Kidney, ureter, and bladder radiograph (Fig. 1) and renal ultrasound revealed two large opacities, one in the bladder, measuring 8.5 cm, and the other in the distal ureter measuring 1.5 cm. She was also found to have a UTI for which she was treated.

## Physical Examination

On examination, the patient was found to have suprapubic tenderness without rebound or guarding. She had no costovertebral or flank tenderness. Bladder scan revealed a postvoid residual of 30 ccs.

## Diagnosis

**FIG. 2.**
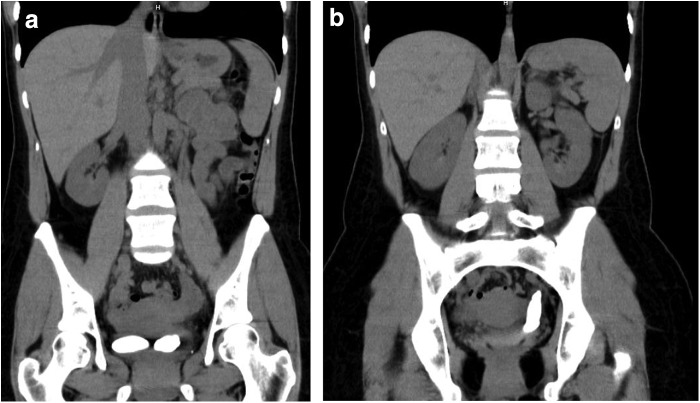
CT scan during work-up revealing **(a)** large bladder stone as well as **(b)** 6 cm stone extending up distal left ureter with duplicated left collecting system.

The patient went on to have a noncontrast and contrast CT scan (Fig. 2), which revealed a 4.4 cm right-sided calculus at the base of the bladder. She was found to have a duplicated system on the left side with a 6.5 cm irregular, cylindrical calculus extending from the left distal upper pole ureter to the intramural tunnel. There was no evidence of hydronephrosis or delayed nephrogram.

## Intervention

The patient was taken to the operating room and underwent a cystoscopy with laser lithotripsy of large bladder calculus. The bladder mucosa was appearing healthy and the ureteral orifices were orthotopic with a patulous left ureteral orifice. A semirigid ureteroscope was advanced up the left ureter and a large stone was noted immediately proximal to the ureteral orifice. Approximately 2 cm from the ureteral orifice, the ureter divided and there was only a small amount of stone in the lower pole ureter. A laser was used to clear the stone from the lower pole ureter. The remainder of the stone was partially fragmented; however, because of the large stone burden, it could not be cleared endoscopically. A Double-J stent was left in the upper pole ureter.

**FIG. 3.**
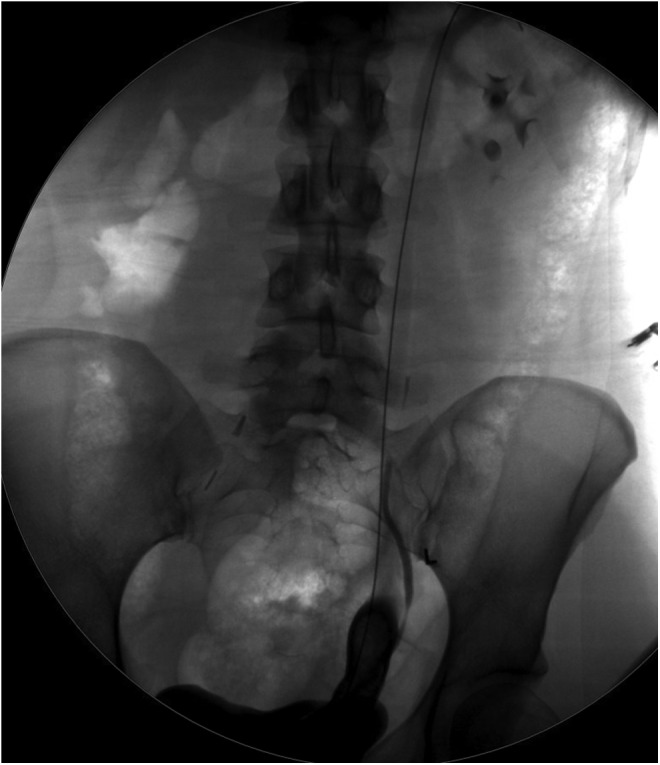
Retrograde pyelogram showing distal ureteral filling defect with dilated upper pole ureter proximal to stone, lower pole stone exiting medially without dilation.

The patient returned for a follow-up procedure 3 months later. A semirigid ureteroscope was effectively advanced into the distal left ureter; however, after prolonged laser lithotripsy, it became clear that the magnitude of stone was not amenable to the endoscopic approach. A retrograde pyelogram was performed (Fig. 3), which showed a normal caliber ureter to the lower pole moiety and dilated upper moiety ureter extending proximally from the edge of the ureteral calculus. A stent was placed into the upper pole moiety and the procedure was terminated.

She then went on to have an MAG-3 lasix renogram that revealed a split function of 44% from the right kidney, 56% from the left kidney, and good function of the upper pole moiety. There was normal T½ clearance bilaterally.

**FIG. 4.**
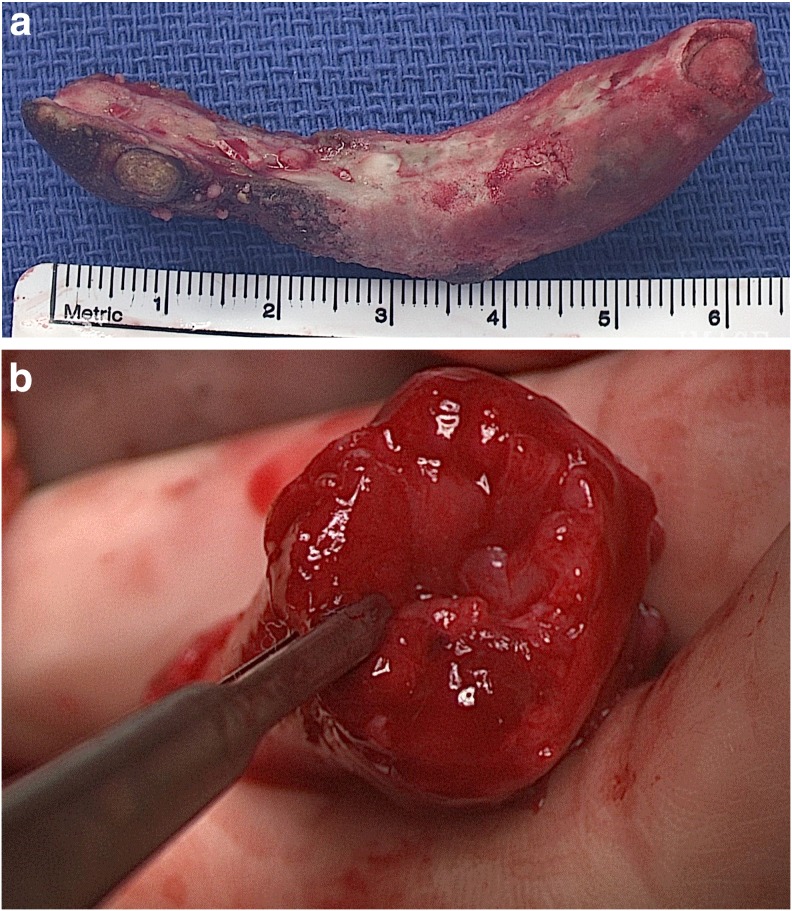
**(a)** Six centimeter stone removed from distal ureter **(b)** cross section of cut duplicated ureter after incision of common ureteral wall, dilated upper pole moiety ureter on the left with DeBakey in lumen.

Four months later the patient returned to the operating room for an open left ureterolithotomy, ureteroscopy with laser lithotripsy of encrusted stent, and common sheath ureteroneocystostomy. Intraoperatively, a 12 g left distal ureteral stone (Fig. 4a) was removed *in toto* from the ureter. The previously placed ureteral stent was found to be encrusted, so a flexible cystoscope was advanced through the transected ureter to the upper pole moiety and laser lithotripsy was performed with effective stent removal. A common sheath reimplant was accomplished by transecting the ureter just proximal to where the calculus had been embedded. The common wall between the distal aspects of the two ureters was transected (Fig. 4b), creating an adequate common lumen that was then reimplanted into the left dome of the bladder through the Wallace technique. A stent was placed into each of the left renal moieties.

## Follow-Up

The patient was discharged home uneventfully on postoperative day 2. One week later she returned to the ED with left flank pain. A CT at that time showed stents in good position without hydronephrosis or evidence of urinary extravasation. She was discharged shortly thereafter on antibiotics for a UTI.

## Outcomes

In the age of rapidly advancing technology, we are increasingly willing to push the envelope with endoscopic management of ureterolithiasis. Owing to increased efficiency and efficacy, percutaneous nephrolithotomy is recommended as first-line treatment for >2 cm renal stone burden^1^; however, no such cutoff exists for escalating procedural invasiveness for large ureteral calculi. Endoscopic approaches for ureteral stones include retrograde ureteroscopy, antegrade ureteroscopy, and laparoscopic or robotic ureterolithotomy. Laparoscopic ureterolithotomy has been found to have equivalent outcomes with shorter hospital stays and better pain control than open procedures,^2^ and thus should be considered first-line therapy for large ureteral calculi.

However, as this case shows, not all stones should be treated endoscopically. For ureteral stones, endoscopic failure has been associated with stone burden >3 cm^2^ and initial open approach should be utilized for stones >5 cm^2^.^3^ For our patient, robot-assisted laparoscopy was considered, however, not favored because of the complexity of the case. In addition to an ureterolithotomy, she required repair of her duplicate ureter and reimplantation. Incidentally, she required ureteroscopy and lasering of an encrusted stent that would not have been possible in a laparoscopic setting.

The current case demonstrates that despite advances in endoscopic technologies, there is still an important role for open surgical management of certain ureteral stones. In planning the surgical approach to stone management, stone burden and location as well as patient anatomy should be carefully considered. Although we are continually pushing the boundaries of endoscopic treatment, open surgery is occasionally the most effective approach.

## Abbreviations Used

CT: computed tomography
ED: emergency department
UTI: urinary tract infection
